# Adjuvant Fuzheng Huayu Capsule Reduces the Incidence of Hepatocellular Carcinoma in Patients with Hepatitis B-Caused Cirrhosis

**DOI:** 10.1155/2020/8826091

**Published:** 2020-10-29

**Authors:** Ke Shi, Yao Liu, Xiaojing Wang, Yuxin Li, Qun Zhang, Ying Hu, Chongping Ran, Yunyi Huang, Jie Hou, Xianbo Wang

**Affiliations:** ^1^Center of Integrative Medicine, Beijing Ditan Hospital, Capital Medical University, Beijing, China; ^2^Department of Gastroenterology, Dongzhimen Hospital, Beijing University of Chinese Medicine, Beijing, China

## Abstract

**Aim:**

Fuzhenghuayu (FZHY) capsule can inhibit the progression of cirrhosis. This study explored whether FZHY can reduce the incidence of hepatocellular carcinoma (HCC) in patients with hepatitis B-caused cirrhosis (HBC) undergoing antiviral therapy.

**Methods:**

A retrospective review of 842 patients with HBC between 2011 and 2015 was performed, including 270 treated with FZHY combined with nucleos (t) ide analogues (NAs) and 572 with NAs alone. The incidence of HCC was compared between the FZHY (*n* = 259) and control (*n* = 259) groups using 1 : 1 propensity score (PS) matching. The incidence of HCC in patients with HBC with different Child-Turcotte-Pugh (CTP) classifications and Toronto HCC risk index (THRI) scores was analyzed using Kaplan–Meier curves.

**Results:**

The 5-year cumulative incidence of HCC before and after PS matching was 151 (17.9%) and 86 (16.6%), respectively. In PS-matched samples, the multivariate Cox proportional-hazards model indicated that the FZHY group demonstrated a significantly lower risk for HCC than the control group (adjusted hazard ratio [aHR] = 0.32, 95% CI 0.19–0.53 *P* < 0.001). The risk of HCC diminished with increased duration of FZHY use. The stratified analysis revealed that the FZHY group, regardless of CTP classification, benefited significantly from FZHY therapy. Patients in the medium- and high-THRI risk groups were the dominant population for FZHY.

**Conclusions:**

FZHY combined with NAs was associated with a significantly lower risk of HCC than NAs alone in patients with HBC, which supports the integration of FZHY with antiviral treatment into clinical practice.

## 1. Introduction

Cirrhosis is the most significant risk factor for the development of hepatocellular carcinoma (HCC). The 5-year cumulative incidence of HCC in patients with hepatitis B virus-caused cirrhosis (HBC) has been reported to be 17% in East Asia and 10% in Western countries [1]. The risk of HCC development in patients infected with hepatitis B virus is 10- to 100-fold greater than in uninfected individuals [2]. In China, more than 80% of HCC cases are associated with hepatitis B virus infection. The majority of these patients (80–95%) have cirrhosis at the time of diagnosis of HCC [3]. HCC has become the third most common cause of cancer-related death with a 5-year survival rate of approximately 35% [4, 5]. Hence, effective preventive measures and therapies are necessary to slow the progression of cirrhosis to HCC.

Currently, the recommended ﬁrst-line options for the treatment of HBC are entecavir (ETV) or tenofovir (TDF) [6]. Studies comparing ETV or TDF with older NAs did not observe a difference in HCC risk reduction between the agents [7, 8]. Studies have found that patients treated with ETV remain at considerable risk for developing HCC despite long-term viral suppression [9]. Some studies reported that the annual incidence of HCC ranges from 0.9% to 5.4% in patients with HBC treated with ETV or TDF [10]. These data indicate that the risk for HCC can be reduced but not eliminated, probably due to risk factors that cannot be modified by antiviral therapy.

In China, traditional Chinese medicine (TCM) has been used to treat chronic liver diseases for centuries [11] and is now a component of alternative therapies and is still used extensively. The severity of liver fibrosis is another risk factor for HCC [12]. Fuzheng Huayu (FZHY) capsule, an effective antifibrosis TCM, has been investigated in both animal experiments [13, 14] and clinical trials [15] for several years. FZHY has demonstrated beneﬁts in patients with cirrhosis, who have experienced significant improvement in liver function and quality of life through decreased collagen synthesis, increased degradation of the extracellular matrix, and reversal of hepatic fibrosis [16]. Song et al. showed that compared with placebo, FZHY could improve clinical symptoms in patients with HBC [15]. A previous study also indicated that the use of TCM is related to decreased HCC risk in patients with chronic hepatitis B [17]. However, few long-term population-based studies have compared the combined application of FZHY and NAs with NAs alone on HCC development among patients with varying clinical stages of HBC [15, 17]. Additionally, it remains unclear whether FZHY can prevent and/or reduce the incidence of HCC.

Accordingly, we aimed to investigate whether FZHY combined with antiviral therapy can reduce the incidence of HCC in patients with HBC and to analyze the role of FZHY in different populations to provide important clinical evidence supporting adjuvant therapy.

## 2. Materials and Methods

### 2.1. Research Subjects

**Figure 1:**
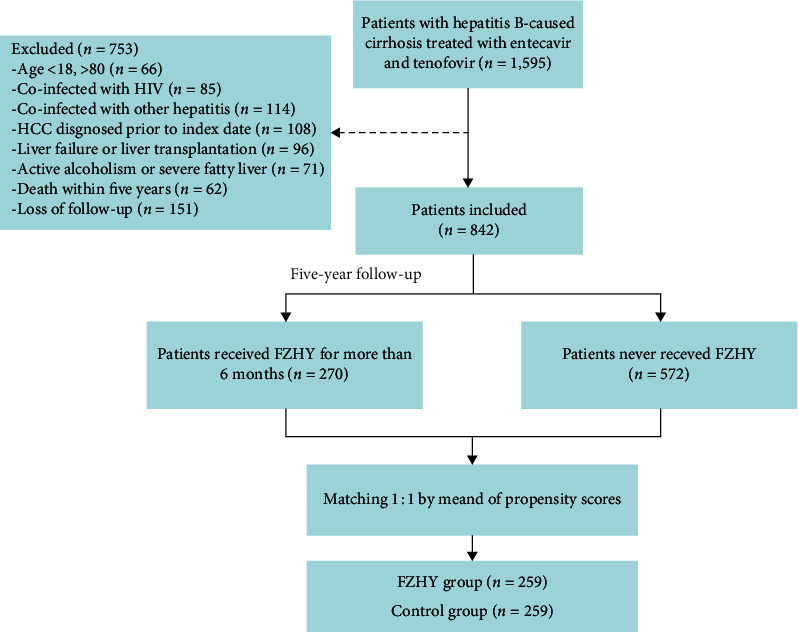
Flowchart of the enrollment of patients with hepatitis B-caused cirrhosis.

We retrospectively enrolled 1,595 patients diagnosed with HBC at the Beijing Ditan Hospital of Capital Medical University (Beijing, China) between January 2011 and January 2015. Patients diagnosed with HBC for the first time, aged >18 and <80 years, and undergoing antiviral treatment with ETV or TDF were included. Individuals infected with human immunodeficiency virus, those with other hepatitis infections (including A, C, D, and E), those with HCC diagnosed before the baseline date, those who underwent liver failure or liver transplantation, those with active alcoholism or severe fatty liver, and those who died within 5-year or lost follow-up were excluded. A total of 842 patients with compensated and decompensated cirrhosis were eventually included. Of them, 270 received FZHY for more than 6 months and 572 did not receive FZHY. Patients were divided into two cohorts according to whether they received combined FZHY (FZHY group, *n* = 259) or no combined FZHY (control group, *n* = 259) by 1:1 PS-matched analysis (Figure 1). FZHY group was defined as the administration of at least one course of FZHY (≥6 months) based on antiviral therapy. This study protocol conformed to the ethical guidelines of the Declaration of Helsinki and was approved by the ethics committee of the hospital.

### 2.2. Clinical Definitions

The baseline date was defined as the date of the ﬁrst diagnosis of cirrhosis in the hospital. The endpoint of this study was the new diagnosis of HCC or the end of the 5-year follow-up. Compensatory cirrhosis was diagnosed as follows: (1) pathological (F4 on biopsy); (2) esophageal varices on endoscopy, exclusion of noncirrhotic portal hypertension; (3) In the absence of histological, endoscopic, two of three criterions should be met:① ultrasonography, computed tomography, or magnetic resonance imaging result indicated imaging changes in liver morphology, such as nodules in the hepatic parenchyma and serrated change on the liver surface; ② platelet count <100 × 10^9^ cells/L, without other causes; ③ serum albumin <35.0 g/L, international normalized ratio >1.3, or prothrombin time prolonged >3 seconds. The diagnosis of decompensated cirrhosis is based on cirrhosis with portal and venous hypertension complications and/or liver dysfunction. (1) Having the diagnosis basis of cirrhosis; (2) having the complications related to portal hypertension, such as ascites, esophageal, and gastric varices bleeding, hepatic encephalopathy [18]. HCC was diagnosed by standard histological and/or compatible radiological findings. Diagnosis is based on imaging techniques obtained by multiple detector CT scan or dynamic contrast-enhanced MRI. The diagnosis can be established if the typical vascular hallmarks of HCC (hypervascularity in the arterial phase with washout in the portal venous or delayed phase) are identiﬁed in a nodule of > 1 cm diameter using one of these two modalities [2]. Virological response (VR) was deﬁned as an undetectable HBV DNA load at the time of HCC development or at the end of follow-up. Routine laboratory investigations, including alpha-fetoprotein and radiological examination, were performed every 3–6 months.

### 2.3. Study Medications

FZHY refers to the herbal extraction, which comprises Danshen (Radix salvia miltiorrhizae), Chongcao (Cordyceps), Taoren (Semen persicae), Jiaogulan (Gynostemma pentaphyllum), Songhuafen (Pollen pini), and Wuweizi (Fructus schisandrae chinensis) (Table 1). All ingredients of FZHY have been approved by the State Food and Drug Administration (SFDA) of China. FZHY capsule is suitable for patients with chronic hepatitis B hepatic fibrosis.

From the above six herbs, Dan shen, Taoren, and Jiaogulan were taken, and they were decocted twice and water was added 10 times and 8 times, respectively (the first time for 2 hours, and the second time for 1.5 hours). Then, the filtrate was concentrated to a relative density of about 1.20 (50°C–55°C) and cooled, and then ethanol was added under mixing to make the alcohol content to 70%. They were then allowed to settle, and the supernatant was taken. After filtration, the filtrate was concentrated to a relative density of 1.1–1.2 (50°C–55°C) and allowed to settle. Further, Chongcao and Wuweizi were taken, and the mixture was heated and refluxed twice and 70% ethanol was added 10 times and 8 times, respectively (the first time for 1.5 hours, and the second time for 1 hour), and the reflux was then combined and filtered. The filtrate was concentrated to a relative density of 1.1–1.2 (50°C–55°C) and kept. Then, Songhuafen was soaked in 50°C 2 times and 50% ethanol was added 10 times and 8 times, respectively (the first time for 4 hours, and the second time for 2 hours), and then it was combined to the extract and concentrated to a relative density of 1.1–1.2 (50°C–55°C). It was then combined with the above two spare concentrates and dried, and a proper amount of auxiliary materials was then added, mixed, crushed into powder, and placed into a capsule (0.3 g/capsule). Z20020073 represents the approval number of national medicine permission number (NMPN) of FZHY capsule (Huanghai Pharmaceutical Co., Ltd., Shanghai, China). The total daily dose of FZHY (0.3 g per capsule) was 15 capsules, administered orally 3 times per day after meals.

### 2.4. Statistical Analysis

Statistical analysis was performed using SPSS version 25 (IBM Corporation, Armonk, NY, USA). A two-sided *P* < 0.05 was considered statistically significant. Normally distributed continuous variables were compared using the *t*-test while the Mann–Whitney *U* test was used for nonnormally distributed variables. Categorical variables were analyzed using the chi-squared test or Fisher's exact test, as appropriate. The crude and adjusted hazard ratio (HR and aHR, respectively) and corresponding 95% conﬁdence interval (CI) were computed and interpreted accordingly. The cumulative incidence of HCC was computed using the Kaplan–Meier curve analysis, and the log-rank test was used to test the difference in the incidence of HCC between groups. Analysis stratiﬁed according to FZHY use was performed using the Cox proportional-hazards regression model to assess the relative risk for HCC between the FZHY group and the control group.

To overcome the effects of possible confounders, propensity scores (PS) were calculated using logistic regression based on age, sex, alcohol consumption, diabetes, hypertension, hepatitis B *e* antigen, albumin, total bilirubin, gamma-glutamyl transferase, white blood cell count, platelet count, creatinine, prothrombin activity, international normalized ratio, alpha-fetoprotein, HBV DNA, Child-Turcotte-Pugh (CTP) class, and model for end-stage liver disease. The FZHY group was matched with the control group according to the generated propensity scores using a one-to-one nearest-neighbor caliper of width 0.02.

## 3. Results

### 3.1. Baseline Characteristics

Among 842 patients with HBC treated with either ETV or TDF, 151 developed HCC within 5 years. Before PS matching, 270 (32.1%) received FZHY for ≥6 months during the study period and 572 (67.9%) did not receive FZHY. FZHY group exhibited a higher rate of diabetes, hypertension, total bilirubin, gamma-glutamyl transferase, white blood cell count, prothrombin activity, and alpha-fetoprotein than the control group. In addition, the FZHY group exhibited lower creatine levels (Table 2).

Among the 259-pair PS-matched population, baseline characteristics were balanced between patients in the two groups. The median age of FZHY and control group was 50 (interquartile range [IQR] 42–59) and 49 (IQR 42–58) years, respectively. A total of 86 HCC events occurred among all study participants, including in 60 control groups and 26 FZHY groups. The cumulative 1−, 3− and 5−year rates of HCC were 0.4%, 6.9%, and 10.0% in FZHY group and 5.0%, 18.1%, and 23.2% in control group, respectively. VR was achieved in 93% of the FZHY group and 96% of the control group at 1 year after ETV/TDF therapy and in 98.8% and 99% of those at the end of the follow-up, respectively.

### 3.2. Effect of FZHY on HCC Occurrence

**Figure 2:**
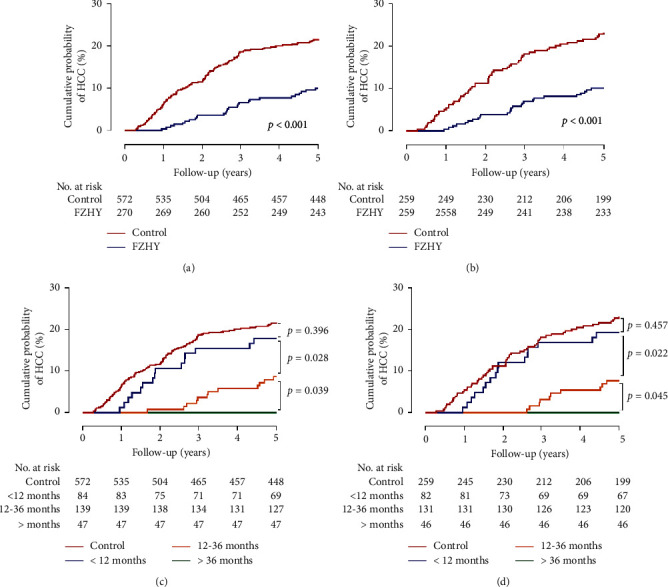
Cumulative incidence of HCC in patients with hepatitis B-caused cirrhosis before (a) and after matching with those with and without FZHY use. (b) Cumulative incidence of HCC in patients before (c) and after matching (d) for different times of FZHY use. HCC: hepatocellular carcinoma; FZHY: Fuzheng Huayu capsule.

Before and after PS matching, the 5-year cumulative incidence of HCC was 151 (17.9%) and 86 (16.6%), respectively. Kaplan–Meier curve analysis revealed that the cumulative incidence of HCC during the 5-year follow-up period was significantly lower in the FZHY group than in the control group, regardless of whether PS matching was performed (all *P* < 0.001) (Figures 2(a) and 2(b)).

FZHY group was divided into three subgroups based on the time of using FZHY: <12, 12–36, and >36 months. The risk of HCC according to the three subgroups was negatively correlated with time of cumulative use. Both before and after PS matching, Kaplan–Meier curve analysis revealed a lower incidence of HCC in the FZHY group who took FZHY for >12 months than in the control group (all *P* < 0.001) (Figures 2(c) and 2(d)). The multivariate Cox proportional-hazards model revealed that after PS matching, the adjusted risk of HCC in FZHY group was signiﬁcantly lower than that in the control group (aHR 0.32, 95% CI 0.19–0.53; *P* < 0.001). FZHY group with 12–36 months of FZHY treatment were at a signiﬁcantly lower risk of HCC (aHR 0.28, 95% CI 0.15–0.54; *P* < 0.001), as were FZHY users with >36 months of FZHY (aHR 0.04, 95% CI 0.01–0.70; *P*=0.028) (Table 3). Thus, the longer the duration of active FZHY therapy, the lower the incidence of HCC.

### 3.3. Subgroup Analysis of the Risk for the Development of HCC

**Figure 3:**
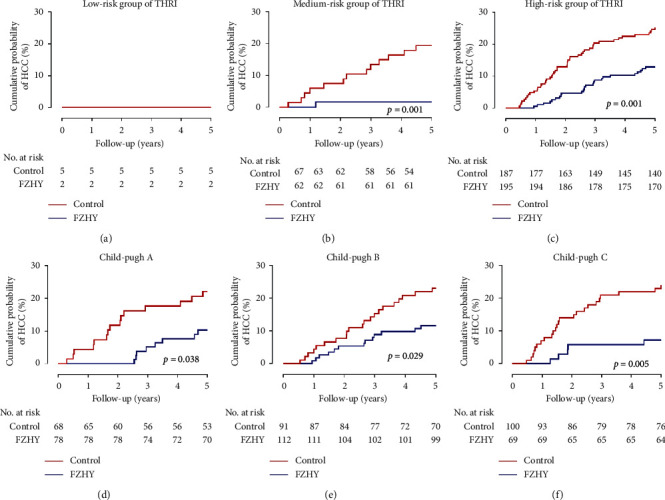
Subgroup analyses of the risk of HCC according to the THRI score and Child-Pugh classification. (a) Low-risk patients; (b) Medium-risk patients; (c) High-risk patients; (d) Child-Pugh A patients; (e) Child-Pugh B patients; (f) Child-Pugh C patients. HCC: hepatocellular carcinoma; THRI: Toronto HCC risk index.

The Toronto HCC risk index (THRI), which is based on age, sex, etiology of liver disease, and platelet count, was developed to predict the risk of HCC in patients with cirrhosis. The score performs well and has been externally validated (Supplementary Table 1) [19]. In this study, most patients (98.6%) were in the medium- (120–240) and high-risk (>240) groups of the THRI score. Only 7 patients were in the low-risk group (FZHY group [*n* = 2], control group [*n* = 5]), none of whom had HCC in 5 years. Regardless of the risk group (i.e., medium or high), the FZHY group demonstrated a significantly lower risk than the control group (medium-risk group, 1.6% versus [vs.] 19.4%; high-risk group, 12.8% vs. 25.1%; all *P*=0.001) (Figures 3(b) and 3(c)) and also had a lower risk of HCC regardless of CTP classification (all *P* < 0.05) (Figures 3(d)–3(f)).

## 4. Discussion

Cirrhosis is a premalignant state with an increased risk for HCC over time [20]. The fibrotic burden is becoming one of the most important factors related to HCC in patients with HBC, especially in those with sustained low viral status [21]. Antifibrosis therapy is an important way to effectively prevent progression to cirrhosis or HCC [11]. There are currently no effective Food and Drug Administration-approved antiﬁbrosis drugs. Thus, many patients use complementary and alternative therapies to protect hepatic function, prevent disease progression, and inhibit the development of HCC.

Accumulating evidence has indicated that FZHY combined with antiviral therapy yields better effects in treating HBV-related cirrhosis, with no serious adverse events [22]. In the present retrospective, population-based cohort study, our results demonstrated that the administration of FZHY was a protective factor for 5-year HCC development in patients with HBC treated with ETV or TDF. The cumulative incidence of HCC in the FZHY group was lower than that in the control group after PS matching (*P* < 0.001). We also found significant relationships between treatment courses and efficiency between the use of FZHY and HCC in patients with HBC. The adjusted HR for FZHY use was 0.66 for <12 months, 0.28 for 12–36 months, and 0.04 for >36 months, indicating that using of FZHY over longer periods would lead to a stronger protective effect.

As a parameter of compensatory liver function, the CTP score has been used to evaluate the efficacy of TCM [23]. Some studies have demonstrated the good therapeutic efficacy of FZHY, which can also improve CTP score in those with HBC [15]. In the present study, FZHY users benefited significantly from antifibrosis therapy, regardless of CTP classification. The THRI has demonstrated good predictive ability for HCC in patients with cirrhosis and has been validated in an external cohort [19]. FZHY group had a lower risk of HCC than the control group, especially in patients with HBC in the medium- or high-risk groups of THRI. Overall, the results of this study add to the evidence supporting the potential role of FZHY in preventing—or, at least, slowing—the development of HCC. These findings may be beneficial to clinicians in providing more effective treatment strategies to improve clinical outcomes.

HCC is strongly associated with liver ﬁbrosis and cirrhosis, with approximately 80–90% of patients with HCC exhibiting underlying ﬁbrosis [24]. Chronic liver injury causes hepatocyte cell death, ﬁbrosis, and, ultimately, the development of HCC [25]. Several studies have shown that a high ﬁbrosis index and liver stiffness, which are indirect measurements of liver ﬁbrosis, are positively correlated with the risk of HCC [26, 27]. Based on proven mechanisms, we analyzed the antifibrotic effect of FZHY in reducing the incidence of HCC, which was as follows: (1) Regulation of the signal transduction pathway: FZHY directly regulates many important pathways, such as the p53 signaling pathway, which correlates with various types of cancer [28]. (2) Regulation of cytokine expression: Transforming growth factor *β*1 (TGF-*β*1) expression level correlated with all stages of disease progression, from fibrosis to cirrhosis and HCC. FZHY remarkably reduces TGF-*β*1 levels [13]. (3) Inhibition of inﬂammatory response: Chronic inflammation initiates ﬁbrosis and cancer. FZHY inhibits the expression of tumor necrosis factor *α* (TNF*α*) and interleukin 6 (IL-6) [14]. (4) Upregulation of the tumor suppressor gene and downregulation of oncogene expression: The previous study has shown that FZHY reduces the expression of Bax, increases the expression of Bcl-2, and decreases the apoptosis of hepatocytes [29]. (5) Inhibition of angiogenesis: Vascular endothelial growth factor (VEGF) is an important factor in tumor growth. It is reported that FZHY can reduce the expression of HIF-1*α*, VEGF, and its receptor [16]. (6) The anti-HCC effects of FZHY may also be due to its regulation of the immune system [30]. Together, these actions may reduce the risk of fibrosis-related carcinogenesis.

To the best of our knowledge, this is the first observational study to investigate the effectiveness of FZHY in patients with multiple clinical stages of HBC by providing real-world evidence. There were, however, a few limitations to our study, the first of which was its retrospective, single-center cohort design, which may have introduced bias in calculating differences in clinical characteristics between the FZHY and the control groups. To address these confounders, patients were matched according to the PS score to eliminate confounding factors. Further prospective randomized controlled trials aiming to assess FZHY for the prevention of HCC development in patients with HBC are warranted. Second, patients may sometimes miss taking FZHY during the follow-up period. However, the FZHY group took medicine for at least 6 months (maximum, 60 months; median, 20 months). The influence on the study is minimal and may not have weakened the effect of FZHY. Third, alcohol consumption is an important factor in the development of HCC. In our study, it was not possible to accurately define the amount of alcohol consumed or to determine whether the patients abstained. It is necessary to reduce chronic exposure to hepatotoxic agents, such as alcohol, to maximize the cancer-preventive effects of antiﬁbrosis TCM. Finally, although only a few (24.1%) patients had previously received antiviral therapy, multivariate Cox proportional risk models were used to test the robustness of the results. FZHY group naïve to NAs were at a signiﬁcantly lower risk of HCC (aHR 0.26, 95% CI 0.10–0.66; *P*=0.001), as were FZHY users who had previously received NA treatment (aHR 0.43, 95% CI 0.26–0.69; *P*=0.005).

## 5. Conclusion

The results of this 5-year follow-up cohort study suggested that FZHY plays an important role as an adjunct therapy to mitigate the development of HCC in patients with HBC. Further prospective cohort studies from multiple centers, however, are needed to verify this conclusion.

## Figures and Tables

**Table 1:** The ingredients contained in Fuzheng Huayu (FZHY) capsule.

TCM name	Latin name	Chinese name	Picture	Weight (g)
FZHY	Radix salvia miltiorrhizae	Danshen	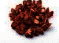	666
	Cordyceps	Chongcao	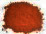	334
	Semen persicae	Taoren	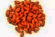	166
	Gynostemma pentaphyllum	Jiaogulan	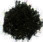	500
	Pollen pini	Songhuafen	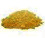	166
	Fructus schisandrae chinensis	Wuweizi	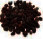	166

**Table 2:** Clinical characteristics of patients with hepatitis B-caused cirrhosis before and after propensity score matching.

Variable	Before propensity matching			After propensity matching		
	TCM user (*n* = 270)	Non-TCM users (*n* = 572)	*P* value	TCM user (*n* = 259)	Non-TCM users (*n* = 259)	*P* value
Age, years	50.0 (42.0, 59.0)	50.0 (42.0, 58.0)	0.525	50.0 (42.0, 59.0)	49.0 (42.0, 58.0)	0.234
Male sex, *n* (%)	191 (70.7%)	393 (68.7%)	0.550	184 (71.0%)	168 (64.8%)	0.132
Family history of HCC, *n* (%)	19 (7.0%)	33 (5.8%)	0.476	18 (6.9%)	16 (6.2%)	0.723
Smoking, *n* (%)	61 (22.6%)	128 (22.4%)	0.944	58 (22.4%)	58 (22.4%)	1.000
Alcohol consumption, *n* (%)	41 (15.2%)	85 (14.9%)	0.984	39 (15.1%)	39 (15.1%)	1.000
Diabetes, *n* (%)	57 (21.1%)	85 (14.9%)	0.024	53 (20.5%)	49 (18.9%)	0.659
Hypertension, *n* (%)	55 (20.4%)	80 (13.9%)	0.018	50 (19.3%)	44 (16.9%)	0.494
Ascites, *n* (%)	46 (17.0%)	77 (13.4%)	0.170	45 (17.3%)	41 (15.8%)	0.637
Hepatic encephalopathy, *n* (%)	14 (5.2%)	18 (3.1%)	0.149	14 (5.4%)	10 (3.9%)	0.403
Gastroesophageal varices, *n* (%)	69 (25.5%)	137 (23.9%)	0.613	65 (25.1%)	81 (31.3%)	0.118
HBeAg positivity, *n* (%)	143 (52.9%)	296 (51.7%)	0.818	139 (53.6%)	126 (48.6%)	0.206
CTP score, *n* (%)	8.0 (6.0, 10.0)	8.0 (6.0, 10.0)	0.837	8.0 (6.0, 10.0)	8.0 (6.0, 10.0)	0.130
MELD score, *n* (%)	10.3 (8.2, 13.2)	10.3 (8.3, 12.9)	0.822	10.3 (8.3, 13.2)	10.7 (8.4, 13.6)	0.573
Alanine aminotransferase, U/L	37.9 (24.1, 80.2)	36.4 (24.0, 76.5)	0.744	37.7 (24.1, 78.3)	37.0 (27.2, 114.5)	0.139
Aspartate aminotransferase, U/L	43.3 (30.8, 76.7)	42.9 (29.1, 85.3)	0.622	43.3 (31.5, 76.5)	46.5 (31.9, 111.0)	0.132
Total bilirubin, *μ*mol/L	27.1 (18.6, 44.8)	23.7 (14.6, 39.7)	0.004	27.2 (18.9, 44.6)	29.5 (16.7, 48.0)	0.838
Albumin, g/L	32.2 (27.9, 37.1)	32.2 (27.7, 37.5)	0.798	32.4 (27.9, 37.0)	32.2 (27.4, 36.4)	0.329
Gamma-glutamyl transferase, U/L	50.3 (24.7, 90.9)	35.7 (20.3, 83.9)	0.010	50.0 (24.9, 90.8)	38.5 (20.8, 85.1)	0.102
White blood cell count, ×10^9^/L	4.2 (3.2, 5.4)	3.6 (2.6, 5.1)	0.001	4.0 (3.1, 5.3)	3.8 (2.7, 5.7)	0.278
Platelets, ×10^9^/L	71.0 (50.4, 101.0)	68.0 (47.2, 99.0)	0.715	71.0 (50.0, 101.0)	69.8 (51.8, 101.0)	0.731
Creatinine, *μ*mol/L	64.0 (53.0, 73.4)	65.9 (56.3, 75.0)	0.027	64.4 (53.5, 73.9)	63.0 (55.0, 72.0)	0.737
Blood urea nitrogen, mmol/L	5.2 (4.2, 6.8)	5.8 (4.1, 6.9)	0.644	5.2 (4.2, 6.9)	5.2 (3.8, 6.8)	0.367
International normalized ratio	1.2 (1.1, 1.3)	1.2 (1.1, 1.4)	0.673	1.2 (1.1, 1.3)	1.2 (1.1, 1.3)	0.726
Prothrombin activity,%	61.0 (50.0, 76.0)	60.0 (48.0, 68.0)	0.021	61.0 (50.0, 76.0)	61.0 (50.0, 73.0)	0.811
Alpha-fetoprotein, ng/ml	10.0 (4.0, 64.4)	6.4 (3.2, 33.0)	0.006	10.0 (4.0, 63.5)	9.0 (4.2, 47.9)	0.926
HBV DNA, log_10_IU/ml	4.2 (2.7, 5.7)	3.8 (2.7, 5.6)	0.118	4.1 (2.7, 5.7)	4.0 (2.7, 5.7)	0.881

Data are presented as *n* (%) or median (interquartile range). CTP: Child-Turcotte-Pugh; MELD: Model for End-Stage Liver Disease.

**Table 3:** Risk of hepatocellular carcinoma according to the cumulative use of FZHY among patients with hepatitis B-caused cirrhosis.

No. of FZHY days	Total	HCC	Hazard ratio (95% CI)			
	(*n* = 518)	(*n* = 86)	Crude^†^		Adjust^*∗*^	
Control group	259	60	Reference		Reference	
FZHY group	259	26	0.39 (0.25–0.62)	*P* < 0.001	0.32 (0.19–0.53)	*P* < 0.001
<12 months	82	15	0.77 (0.44–1.37)	*P*=0.381	0.66 (0.35–1.24)	*P*=0.197
12–36 months	131	11	0.32 (0.18–0.62)	*P*=0.001	0.28 (0.15–0.54)	*P* < 0.001
>36 months	46	0	0.04 (0.01–0.70)	*P*=0.028	0.04 (0.01–0.70)	*P*=0.028

Data are presented as hazard ratios (95% confidence intervals). ^*∗*^. Crude HR represents relative hazard ratio. ^*∗*^. Adjusted HR represents multivariate-adjusted hazard ratio: age, sex, drinking, total bilirubin, albumin, white blood cell count, platelets, creatinine, blood urea nitrogen, international normalized ratio, prothrombin activity, alpha-fetoprotein, and HBV DNA. FZHY: Fuzheng Huayu capsule.

## Data Availability

The data used to support the findings of this study are available from the corresponding author upon request.

## Abbreviations

FZHY: Fuzheng Huayu capsule
TCM: Traditional Chinese medicine
HCC: Hepatocellular carcinoma
HBC: Hepatitis B-caused cirrhosis
NAs: Nucleos (t)ide analogues
PS: Propensity score
CTP: Child-Turcotte-Pugh
THRI: Toronto HCC risk index
aHR: Adjusted hazard ratio
ETV: Entecavir
TDF: Tenofovir
CI: Conﬁdence interval.

## Supplementary Materials

Supplementary Materials Supplemental Table 1: components of the Toronto HCC Risk Index.
