# Supplementary material for: Adjuvant Fuzheng Huayu Capsule Reduces the Incidence of Hepatocellular Carcinoma in Patients with Hepatitis B-Caused Cirrhosis
Source: Evid Based Complement Alternat Med. 2020 Oct 29;2020:8826091. doi: 10.1155/2020/8826091 (PMC7644307; doi:10.1155/2020/8826091)
Supplement: Supplementary Materials — Supplemental Table 1: components of the Toronto HCC Risk Index. [file 8826091.f1.docx]

Supplementary table 1. Components of the Toronto HCC Risk Index.

| Risk Factor | Score |
| --- | --- |
| Age |  |
| < 45 | 0 |
| 45–60 | 50 |
| >60 | 100 |
| Etiology |  |
| Autoimmune | 0 |
| HCV SVR | 0 |
| Other | 36 |
| Steatohepatitis | 54 |
| HCV | 97 |
| HBV | 97 |
| Gender |  |
| Female | 0 |
| Male | 80 |
| Platelets |  |
| >200 | 0 |
| 140–200 | 20 |
| 80–139 | 70 |
| <80 | 89 |
| Total | 0–366 |

HBV, Hepatitis B virus; HCV, Hepatitis C virus; SVR, sustained virologic response.
